# Balancing Complex Signals for Robust Predictive Modeling

**DOI:** 10.3390/s21248465

**Published:** 2021-12-18

**Authors:** Fazal Aman, Azhar Rauf, Rahman Ali, Jamil Hussain, Ibrar Ahmed

**Affiliations:** 1 Department of Computer Science, University of Peshawar, Peshawar 25120, Pakistan; fazalaman@uop.edu.pk (F.A.); ibrar@uop.edu.pk (I.A.); 2 Quaid-e-Azam College of Commerce, University of Peshawar, Peshawar 25120, Pakistan; rehmanali@uop.edu.pk; 3 Department of Data Science, Sejong University, Seoul 05006, Korea

**Keywords:** modern machine learning, classical machine learning, balancing complex signals, outliers

## Abstract

Robust predictive modeling is the process of creating, validating, and testing models to obtain better prediction outcomes. Datasets usually contain outliers whose trend deviates from the most data points. Conventionally, outliers are removed from the training dataset during preprocessing before building predictive models. Such models, however, may have poor predictive performance on the unseen testing data involving outliers. In modern machine learning, outliers are regarded as complex signals because of their significant role and are not suggested for removal from the training dataset. Models trained in modern regimes are interpolated (over trained) by increasing their complexity to treat outliers locally. However, such models become inefficient as they require more training due to the inclusion of outliers, and this also compromises the models’ accuracy. This work proposes a novel complex signal balancing technique that may be used during preprocessing to incorporate the maximum number of complex signals (outliers) in the training dataset. The proposed approach determines the optimal value for maximum possible inclusion of complex signals for training with the highest performance of the model in terms of accuracy, time, and complexity. The experimental results show that models trained after preprocessing with the proposed technique achieve higher predictive accuracy with improved execution time and low complexity as compared to traditional predictive modeling.

## 1. Introduction

Data mining is the process to extract interesting patterns from structured and unstructured data. More precisely, organizations obtain valuable patterns from large datasets for better decision making. Among other pattern revealing techniques, predictive modeling is used to foresee future outcomes [1]. During predictive modeling, a dataset is split into three sets; the training set, used to build the model, the validation set, used to fine tune the model, and the test set, used to check the predictive accuracy of the built model. Statisticians believe that data can always be decomposed into signals and noise [2], though researchers try to train a model on maximum signals (instances) of the training set to reduce the bias errors. However, they try to avoid training the model on outliers to reduce the variance errors. This approach usually maintains a balance between the bias and variance errors. The formulation for computing the total error [3] of the model is presented in Equation (1).


(1) $$TotalError={\left(Bias\right)}^{2}+Variance+IrreducibleError$$

The irreducible error occurs due to the presence of noise and outliers in the data. Various techniques [4,5,6,7,8,9,10] have been used for the removal or winsorization of the outliers from the dataset to improve the modeling accuracy. However, Abraham et al. [2] suggest that if the interpolated classifier deals with the outliers locally, their adverse impact on the prediction may be minimized. Recently, Mikhail et al. [11] claimed that additional training of the model after an interpolation point leads to a modern interpolating regime, where the accuracy of the model once again starts improving. This claim has been proved by the double descent curve [11], in which the training risk becomes minimum while the testing risk remains maximum at the interpolation point. The testing risk, however, starts dropping once again by increasing the complexity of the model, which results in good performance on the unseen data after the interpolation point.

In the classical approach, the complete removal of outliers from training datasets may cause the loss of important information. Outlier values notably vary from the data distribution. Although, outliers may have an adverse impact on the performance of models, they may also contain important information, and hence, their removal is not always suggested [12]. On the other hand, in modern machine learning, outliers are regarded as complex signals and are not removed from the training datasets. They are rather considered during the training process. However, their adverse impact on the model’s performance may be reduced by increasing the complexity of the model.

In the modern interpolation regime, models are overtrained after the interpolation point while their complexity is increased to overcome the effect of the outliers [2].These models consider outliers during the training process, but even being on high complexity levels, they usually fail to achieve the correctness of the classical models. Gaining motivation from this aspect, this work proposes a novel technique to:

- Identify outliers in the dataset along with their impact on the model’s performance that includes predictive accuracy, efficiency, and complexity.
- Perform a trade-off analysis between the inclusion and exclusion of the number of outliers in the training set for computing its impact on the model′s performance.
- Identify and suggest an optimal point at which the maximum number of outliers (complex signals) may be included in the training set with minimum deteriorating impact on the performance of the model.

The rest of the paper is organized as follows: Section 1 critically analyzes the related work. Section 2 explains the proposed Complex Signal Balancing (CSB) technique with the help of algorithms and flowcharts. Section 3 presents the experimental design, setup, and implementation. Section 4 Discussion and Analysis to compares the proposed CSB approach with the state-of-the-art approaches. Section 5 concludes the work with some future directions.

### 1.1. Basic Concepts

This section discusses the basic concepts regarding classical and modern machine learning.

#### 1.1.1. Classical Supervised Machine Learning

Predictive techniques of machine learning are used to build models that can predict future outcomes [1]. Prediction has been one of the most widely used application areas of machine learning for some time. In prediction problems, a given sample of training examples (x^1^, y^1^) (x^n^, y^n^) from R^d^ × R, a predictor $h_{n}\to R^{d}\times R$ is learnt to predict the outcome of an unseen instance. The predictor is a function, h_n_ ∈ H, such that it minimizes the training error and is written using Equation (2) [11].

(2) $$\frac{1}{n}\overset{n}{\underset{i=1}{\sum }}\ell \left(h\left(x_{i}\right),{\;y}_{i}\right)$$

The training error is computed by averaging the loss function, ℓ. For regression, the squared loss is computed in the form $\ell \left(y\prime ,y\right)={\left(y\prime -y\right)}^{2}$, and for classification, the loss function is described as $\;\ell \left(y\prime ,y\right)=1_{\left\{y\prime \neq y\right\}}$, which is also called zero-one loss [11]. The goal of machine learning is to minimize the test error, which is given in the form of Equation (3) [11].

(3) $$E_{\left(x,y\right)}\sim \;P\;\left[\ell \left(h\;\left(x\right),\;y\right)\right]$$

where P is the probability of minimum lost (ℓ) and the predictor, h is applied to the independent variable of point x for predicting  y.

**Figure 1:**
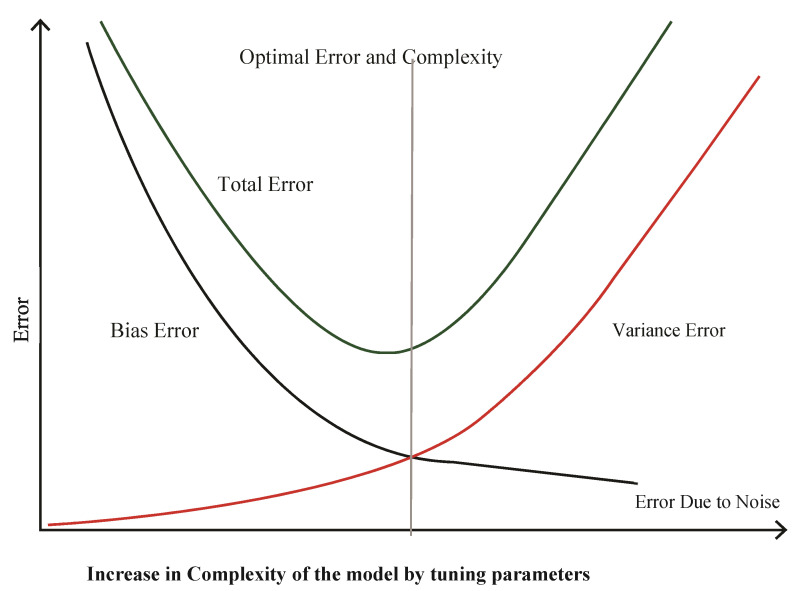
Bias Variance Trade-off. Reprinted with permission from ref. [3]. Singh, S (2018).

Traditionally, in classical machine learning, reduction in test error can be achieved by finding the sweet spot (the point where the model has minimum bias and variance errors) using a bias-variance trade-off [11]. The classical bias-variance trade-off [3] is shown in the following Figure 1. The goal of a well-trained model is to find the point where the model has minimum bias and variance errors. In Figure 1, the model has optimum complexity at the intersection point of the two error lines.

#### 1.1.2. Modern Machine Learning

**Figure 2:**
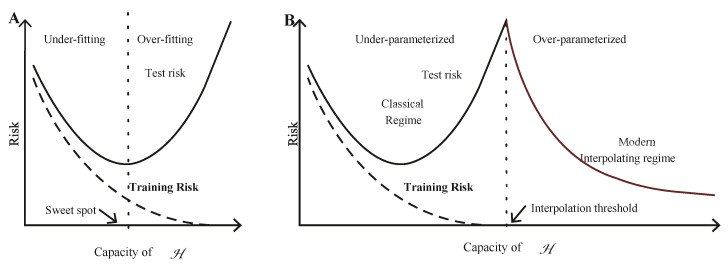
Classical (**A**) and double descent curves (**B**). Reprinted with permission from ref. [11]. Belkin, M et al. (2019).

In the classical machine learning approach, it is believed that when the training set fits, the model reaches to the interpolation point, where the model has minimum bias error and maximum variance error. This state of the model is also called the spiked state. In the modern interpolation regime, a further increase in the capacity of function class H is achieved by extracting more features from the training set to increase the smoothness of the model. This activity further decreases the variance error and the model starts improving performance on unseen data with low true risk (variance error) [11]. Figure 2A,B depict the classical machine learning and double descent error curves, respectively. Figure 2B combines the classical and modern interpolating regime curves used in traditional and modern machine learning, respectively. The training error becomes zero after the interpolation point. Figure 2B depicts that the model’s performance is improved after the interpolation point because of the decrease in training and testing errors [11].

Mikhail et al. [11] has claimed the existence of a double descent curve by providing empirical evidences over different predictive models and datasets. The authors first considered a popular class of non-linear parametric models called Random Fourier Features (RFF) model family H_N_ with N (complex-valued) parameters consisting of functions $h\;:R^{d}\to C$ of the form [11] given in Equation (4).

(4) $$h\left(x\right)=\sum_{k=1}^{N}a_{k}\phi \left(x,v_{k}\right)\;\mathrm{where}\phi \left(x;v\right)=e^{\sqrt{-1_{\left(v,x\right)}}}$$

The data point x is passed through N RFF functions $\phi \left(x,v_{k}\right)$; the function actually computes exponent of the product of x and vector v, i.e., $e^{\sqrt{-1_{\left(v,x\right)}}}$, followed by aggregation ($a_{k}$) of the function′s results. Mikhail et al. [11] empirically demonstrated that the increase in the number of features beyond the interpolation point produces the double decent curve with the improved accuracy of the model. Javier et al. [13] considered the individual variability of the length-at-age using a mixed-effect model, where non-gaussian distributions, such as Student-t, is also considered. The classifier interpolated in this way, with the outliers dealt locally, results in the minimum possible effect of outliers on prediction [2]. Hyper parameter tuning [14,15,16,17,18] is used to improve the predictive accuracy of machine learning algorithms. However, such techniques incur high computational cost.

#### 1.1.3. Outliers

Outlier values are notably different from the normal data distribution. Computer scientists consider the outliers as complex signals [2]. Although such signals may have an adverse impact on the performance of model, their removal is not always legitimate [12] as they may carry important information. There is no consensus on a solid mathematical definition for outliers; however, some statistical tests are available for finding candidate outliers [12]. The main contribution of this paper is to devise a novel technique, Complex Signal Balancing (CSB), for training models with outliers until an acceptable performance for a given dataset is achieved. Models trained using the proposed CSB technique outperform the Modern Machine Learning (MML) models in terms of efficiency and predictive accuracy.

### 1.2. Related Work and Problem Statement

In the literature, there has been much debate on what to do with extreme value observations (outliers) [2]. They represent much smaller or larger values than the majority of data points, and the inclusion of a few outliers may deteriorate the results [19]. A small proportion of the outliers can distract even simple analyses. Simple techniques of *Z-Score* = 3 and ANOVA were used by Jason et al. [20] to remove extreme scores from the dataset, which resulted in reduced errors with significantly improved accuracy. A two phase clustering algorithm for outlier detection was proposed by Jiang et al. [21]. In phase 1, the traditional k-means algorithm is used to split data into outliers and normal data points, placing them in their respective clusters. In phase 2, the minimum spanning tree is used to remove the longest edges and replace the original tree with two newly generated sub trees. The small clusters with fewer nodes are considered as outliers. Their proposed process was applied for anomaly detection and monitoring e-mails, which showed effective results.

Tukey′s schematic boxplot is used as a test for the existence of outliers [4]. Sim et al. [5] recommended a graphical boxplot instead of a commonly constructed boxplot and claimed more precise labeling of the outliers. Dawson et al. [6] suggested that a boxplot is useful for outlier detection but should be used with caution, as at least 30% of samples from normally-distributed data are flagged as containing outliers. Schwertman et al. [7] suggested a method that is used by data analysts for specifying the outlier criteria. The innerfence (1.5 × IQR) is suggested for a normal distribution and is approximately 2.70 standard deviations above and below the mean. Askewness-adjusted outlyingness (AO) is an outlier detection method that was proposed by Hubert et al. [8] for multivariate skewed data. The analysts applied their method on simulated and real data and claimed that the proposed outlier detection method identified the outliers without assumption of symmetry and did not rely on visual inspection.

Shahian et al. [9] proved that outliers lead to undesirable consequences. Hence, scientific, firm, and sound judgments are required to accurately classify outliers for improving healthcare quality. A boosting algorithm, SavageBoost, was proposed by Masnadi et al. [22] that had more resistance to outliers than classical methods, such as AdaBoost and RealBoost [22]. Nyitrai et al. [10] used omission and winsorization techniques for handling outliers. The extreme value identifications were carried out via standardization at the value of two and three standard deviations from the mean. Various predictive methods have been used for detection of outliers in the dataset [23,24,25,26,27]. The authors remarked that neural networks and linear models are sensitive to noise points, whereas decision trees are robust to outliers. They suggest that the performance of multilayer perceptron, discriminate analysis, and logistic regression may be improved by handling outliers. The Random Forests algorithm is a combination of tree predictors and can be used both in classification and regression problems. The generalization error of Random Forests converges with the increase in the number of trees in the forest, which is more robust to noise [28]. Various predictive models have been used in the education field for improving the quality of education at institutions and for predicting student′s academic performance [29,30,31,32,33,34,35,36,37,38,39,40]. In this paper, we use a dataset obtained from the examination section of the University of Peshawar for analysis purposes.

The prevalent literature indicates that researchers have given little attention to properly analyze outliers before their removal from the training dataset. There is a need to analyze a dataset during the preprocessing step for including or excluding the number of outliers in the training dataset for building robust predictive models. In the classical approach, the yardstick to use is 1.5 × IQR or 2.7σ standard deviations from the mean for the identification and removal of outliers from the training dataset. These outliers, however, may carry critical information and should not be removed from the training dataset without proper investigation. Our objective is to include the maximum number of outliers in the training dataset. Hence, the static bar of 1.5 × IQR above or below the mean for outliers needed to be re-evaluated and modified as per the nature of the dataset.

On the other hand, in the MML approach, a predictive model is trained after the interpolation point, and all outliers are considered during the training process [11] because of their significance as complex signals. An issue in the MML approach is the identification of the sweet spot, because predictive models become computationally complex at the interpolation point. Furthermore, early convergence of a predictive model to a sweet spot is needed in the modern interpolation regime to achieve better efficiency. In summary, there is a need to investigate the effect of outliers on the performance of the predictive model, including complexity, efficiency, and accuracy in the classical as well as the modern interpolation regimes. This tradeoff analysis will help to introduce a sweet spot for predictive models in the modern interpolation regime. This study proposes a new CSB technique that may be used as a preprocessing step in the generation of a robust predictive model to overcome the above stated issues.

## 2. Proposed Approach

This research work proposes a preprocessing step to incorporate the maximum number of complex signals in a training dataset for building robust predictive models. One of the challenges in the proposed technique was the identification of outliers. For this purpose, a well-known Tukey′s schematic boxplot concept is used [4]. However, it is a univariate approach and not applicable on a typical dataset involving several attributes. For this purpose, we run a loop and apply Tukey′s approach to a single attribute at a time. Hence, the problem of outlier identification in a dataset having ‘*n*’ attributes is reduced to a single attribute at a time. The proposed approach analyzes the dataset for inclusion or exclusion of outliers (complex signals) in the training dataset, depending on the performance of a predictive model. It sets a dynamic threshold  (1.5+λ)×IQR below or above the mean for the identification and exclusion of outliers from the training dataset, as per the nature of data. The parameter  λ is used as a tradeoff parameter of complex signals by shifting the inner and outer fences. The number of complex signals to be considered in the training dataset increases as we increase the value of λ. The percentage of complex signals and the model performance is determined at the successive value of λ. The proposed technique introduces a new sweet spot in the modern interpolation regime after performing a tradeoff analysis among the percentage complex signals, predictive accuracy, time efficiency, and complexity of the predictive model.

**Figure 3:**
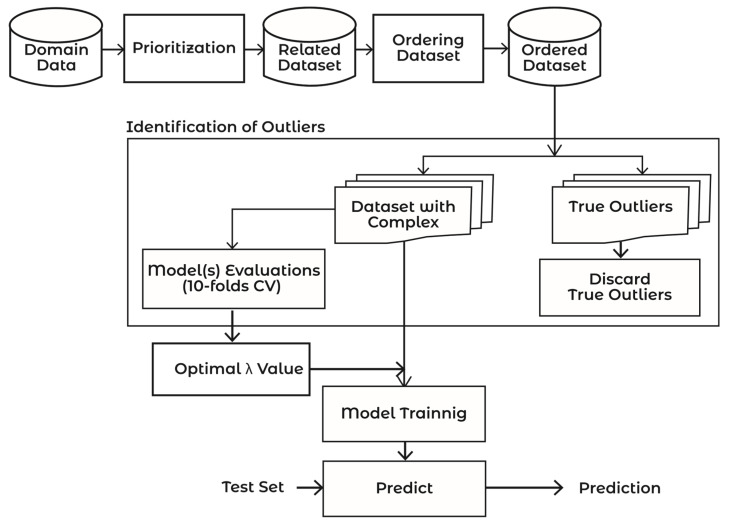
Complex Signals Balancing (CSB) framework.

Figure 3 and Algorithm 1 demonstrate the workings of the proposed approach. The proposed framework consists of the following steps: (1) Prioritization, (2) Ordering, and (3) Outliers Identification. The subsequent sections explain the working of the proposed approach.
**Algorithm 1: Balancing Complex Signals**Begin  inputs:d –PPDS //ordered dataset  *RF*—the learning algorithm$\bar{Q}$ = {e_1_ = *f* − *measure*, e_2_ = *kappa*, e_3_ = accuracy}–the set of evaluation metrics;**output:*Optimal model*****1.** RDS = Boruta(PPDS)**2.** ODS = Order_dataset(RDS)**3.** *For* (λ = 0, λ ≤ 0.5, λ = λ + 0.05)
A.ds = totds; clds = empty; olds = emptyB.(***for k* = 1*ncolumn(ODS)***
a.If(not(factor(ODS[k]) thenb.*classlabl* = *count(ODS[end])*c.***Repeat***i.u = Q3 + (1.5 + λ) * IQR(claslabl, attrbt)ii.l = Q1 − (1.5 + λ) * IQR(claslabl, attrbt)iii.cl = cleandataset(l, u, ODS, claslabl, k)iv.*cs* = complexsignals(l, u, ODS, claslabl, k)v.*clds* = combinedataset(clds, cl)vi.*csds* = combinedataset(csds, cs)vii.*next(classlabl)*d.***Until(end of classlabl)***e*ds* = *clds*f*tot_cs* = combinedataset (tot_cs, csds);g*csds* = *empty*;h*end if*; // end of step i.
C.**End For // step-b**
**4.** Clean_Models_λ = BuildModel(clds, RF, ntrees)**5.** Complex_Models_λ = BuildModel(tot_cs, RF, ntrees)**6.** Results_Clean_ = ResultsClean + addPer (testModel(TestData, Clean_Models_λ,))**7.** End For // step-1**8.** Display_Graphs_ (Result)**9.** *Models_Results* = Clean_Models_λ, Results_Clean_**10.** ${\mathrm{Opt}}_{{\mathrm{Perf}}^{\lambda }}=\frac{{\mathrm{Accuracy}}^{\lambda }\;*100}{\left(perc\_cs^{\lambda }\right)}$**11.** $\mathrm{Retutn}\;({{\mathrm{Opt}}_{\mathrm{Perf}}}^{\lambda })$

In step 1 of Algorithm 1, the pre-processed dataset ($\mathrm{ppds}=f_{1}+f_{2}+f_{3}+\ldots f_{n}$) is passed to the Boruta algorithm for removing unrelated attributes; the resultant dataset is called the Related Dataset ($\mathrm{RDS}=f_{1}+f_{2}+f_{3}+\ldots f_{n}$). In step 2, the resultant RDS is passed to the sorting procedure “Order dataset” for arranging the attributes in the order of their influence on the goal. The resultant dataset is called the Order Dataset $\left(\mathrm{ODS}=f_{1}{}^{p1}+f_{2}{}^{p2}+f_{3}{}^{p3}\ldots +f_{n}{}^{\mathrm{pn}}\right)$, where $f_{1}{}^{p1}$ is the most influential and $f_{n}{}^{\mathrm{pn}}$ is the least influential attribute. In Algorithm 1, the “BuildModel” procedure is called to build a model using the 10-fold cross validation method. In step 6, the model results on a test dataset are saved. In step 9, the model on the optimal value of λ is selected as the optimal model for the dataset.

### 2.1. Prioritization

To achieve the promising results, we identify the most influential attribute over the class label attribute in the entire dataset and subsequently identify outliers in that attribute. This process is repeated for the second influential attribute in the dataset and so on until all outliers are identified. For this purpose, we calculate the Mean Importance (MI) of all attributes and sort them in the descending order of this value.

**Figure 4:**
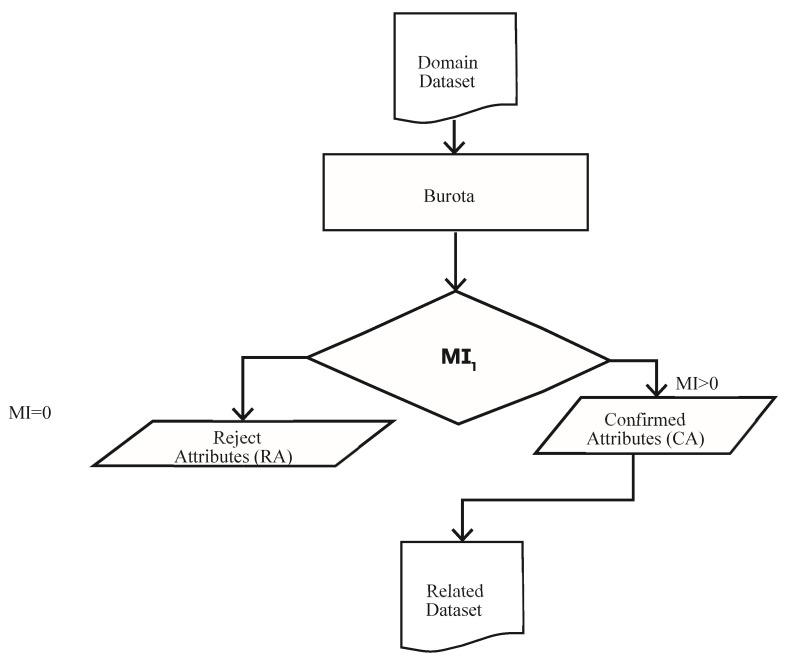
Boruta algorithm for prioritization and selection of attributes.

The MI of the attributes is computed by adopting the feature selection Boruta algorithm [41], which is an improvement on the Random Forest algorithm for variable importance measure and selection [42]. Random Forest uses the Mean Decrease in Accuracy and Mean Decrease in Gini [43]. The variable importance is calculated using the formula given in Equation (5) [43].

(5) $$MI_{j}=\frac{1}{ntree}\overset{ntree}{\underset{t=1}{\sum }}\left(EP_{tj}-E_{tj}\right),$$

where *ntree* denotes the number of trees, *E_tj_* denotes the out of bag error on the tree ‘*t*’ before permuting the values of attribute *X_j_*_,_, and *EP_tj_* denotes the out of bag error on the tree ‘*t*’ after permuting the values of *X_j_* results in the identification of the influential features that largely affect the target variable. The algorithm works on the Random Forest classification method. It iteratively removes the features that are proved irrelevant, i.e., having zero MI. The dataset is passed to the prioritization step in Figure 3, where the Boruta algorithm is used to mark each attribute as “Confirmed” or “Rejected”. The flow of this process is depicted in Figure 4.

### 2.2. Ordering

The next step is ordering, as shown in Figure 3. The main objective of this step is to sort the attributes of the dataset in the order of their importance, as per the target attribute. The output MI of the Boruta algorithm in the previous step is passed to this step as input. The attributes are sorted in descending order according to their MI values. The output of this step is the ordered dataset, given in Equation (6).

(6) $$ODS=f_{1}{}^{p1}+f_{2}{}^{p2}+f_{3}{}^{p3}\ldots +f_{n}{}^{pn}$$

Here, the attributes are arranged in the descending order of their MI values from left to right i.e., $f_{1}{}^{p1}$ is the most influential attribute, having the highest MI, and $f_{n}{}^{pn}$ represents the least influential attribute, having the lowest MI value.

### 2.3. Identification of Complex Signals

Once the dataset is sorted in descending order according to corresponding MI values, it is ready for the identification of outliers in each attribute, separately using Tukey′s approach [4]. The default limits of complex signals in this approach are given below, via Equations (7) and (8).

(7) $$\mathrm{Outer}\;\mathrm{Fence}=Q_{3}+\left(1.5\right)\times \;\mathrm{IQR}$$

(8) $$\mathrm{Inner}\;\mathrm{Fence}=Q_{1}-\left(1.5\right)\times \;\mathrm{IQR}$$

**Figure 5:**
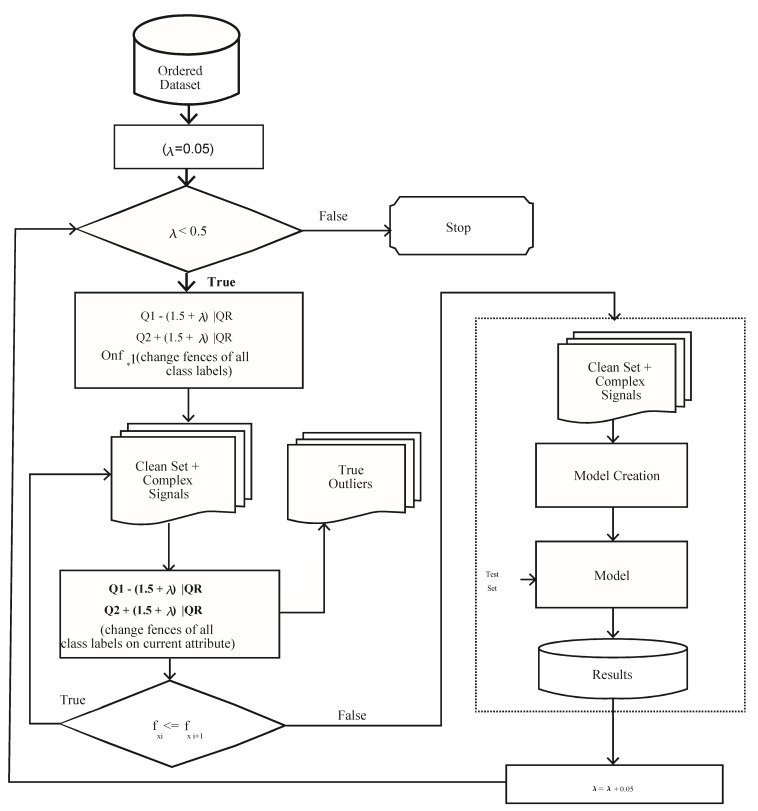
Identification of Complex Signals.

According to this approach, all data points lying above the outer fence or below the inner fence are considered to be complex signals. However, in the proposed approach, these fences are modified as follows, using Equations (9) and (10).

(9) $$\mathrm{Outer}\;\mathrm{Fence}=Q_{3}+\left(1.5+\lambda \right)\times \mathrm{IQR}$$

(10) $$\mathrm{Inner}\;\mathrm{Fence}=Q_{1}-\left(1.5+\lambda \right)\times \mathrm{IQR}$$

where λ is the parameter for modifying inner and outer fences. Different values of λ vary the inner and outer fences, which results in the fluctuation of the number of complex signals to be included or excluded from the training dataset. The proposed algorithm first selects an appropriate λ value, then complex signals are identified based on the first influential attribute, followed by the second influential attribute, and so on. For this fixed value of λ, all irrelevant complex signals are dropped from the dataset and a model is trained on the remaining dataset, as shown in Figure 3. The model’s performance in terms of predictive accuracy, time efficiency, and complexity is checked for this selected value of λ using a 10-fold cross validation technique. The same process is repeated for a new value of λ in subsequent iterations until the value of λ is found for which the model gives the optimal performance. The sweet point is the value of λ for which the maximum number of complex signals is considered to train a model for optimal performance. In other words, an optimum value of λ drops the minimum possible number of complex signals from the training dataset. The process is shown in Figure 5. Equation (11) calculates the optimal value of λ for a model:

(11) $${\mathrm{Opt}}_{{\mathrm{Perf}}^{\lambda }}=\frac{\mathrm{Accurac}y^{\lambda }\times 100}{\left(Perc_{CS^{\lambda }}\right)}$$

where ${\mathrm{Accuracy}}^{\lambda }$ and $Perc_{CS^{\lambda }}$ depict the accuracy and the percentage of complex identified signals for a given value of λ, respectively.

## 3. Experiment Design

The experiments were conducted on three datasets, including the University of Peshawar, MNIST, and Phoneme datasets. The R-Language with R-Studio was used for the implementation purposes. Random Forest library, Performance Analytics, and Caret were used as the simulation tools on a standalone Intel^®^ Core^™^ i5-4300M CPU @ 2.60 GHz system with 4 GB RAM.

### 3.1. Comparison of Results on University of Peshawar Dataset

The real-world indigenous dataset obtained from the University of Peshawar consists of 17,463 records, with fifteen academic features, four socio-economic features, and one demographic feature. The output variable, i.e., class attribute of the dataset, has a possible five values: A, B, C, D and F. A sample of the dataset is shown in Table 1.

The Total credit hours (Tot_ch), Batch (Batch), and Roll Number (Roll_No), being identified as less influential on the target attribute, were removed during the initial preprocessing step. The production data of the University of Peshawar was taken for experimentation purposes and consist of 12,226 records for training and 5237 records for testing. Predictive models were built based on the proposed CSB and MML approaches. Students′ grades were predicted from models of both approaches and compared on the basis of different performance evaluation metrics. The optimal value of λ for this dataset was selected to be 0.2.

**Figure 6:**
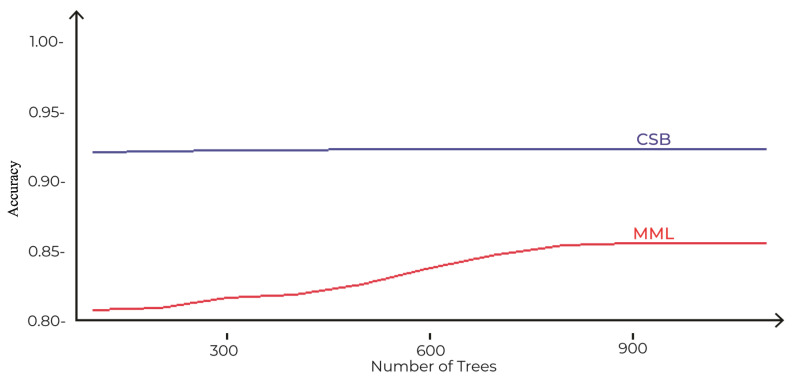
Comparison of CSB and MML approaches based on Accuracy using the UoP Dataset The execution time of the CSB model is less than the MML model, as shown in Figure 7. As the number of trees increases, the gap of execution time between the two models also increases, showing that the CSB model is more efficient in terms of execution time compared to the MML model. The optimal point where CSB achieved maximum 93% accuracy is at only 100 trees. The comparison of the CSB and MML on Kappa and F-Measure are prescribed in Figure 8 and Figure 9, respectively.

The comparison of accuracies of MML and the proposed CSB models based on different number of trees as tuning parameters is shown in Figure 6. The maximum accuracy achieved by the MML model is 89%, with 900 trees using the Random Forest algorithm. However, the CSB model achieved 93% accuracy with just 100 trees. This shows that the proposed approach has achieved higher accuracy with less complexity.

### 3.2. Results and Comparison on MNIST Dataset

In the MNIST dataset, 60,000 records were used as a training and 10,000 records as a testing set of handwritten digits. Predictive models were built based on the proposed CSB approach and MML approach [11]. The model built on the MML approach results in training and testing errors of 32% and 35%, respectively, for a single tree of 10 nodes, as shown in Table 2. The training and testing errors are further reduced to 9% and 10%, respectively, after increasing the number of nodes to 2000 with 20 trees. This shows that both training and testing errors are reduced after the interpolation point at a cost of the increased complexity of the model. On the other hand, the model built using the proposed CSB approach incurred the training and testing errors of 24% and 26%, respectively, for a single tree of 10 nodes. The training and testing errors are drastically reduced to 3% and 6%, respectively, after increasing the number of nodes to 2000 with 20 numbers of trees. These results show that, on average, there is an 8% reduction in training and testing errors with the proposed technique compared to the MML technique. The optimal value of λ for this dataset is found to be 0.1. Figure 10 depicts these results graphically.

**Figure 7:**
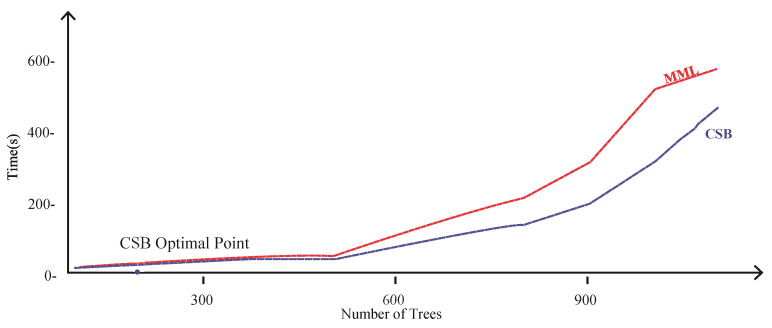
Comparison of CSB and MML approaches based on execution time using UoP Dataset.

**Figure 10:**
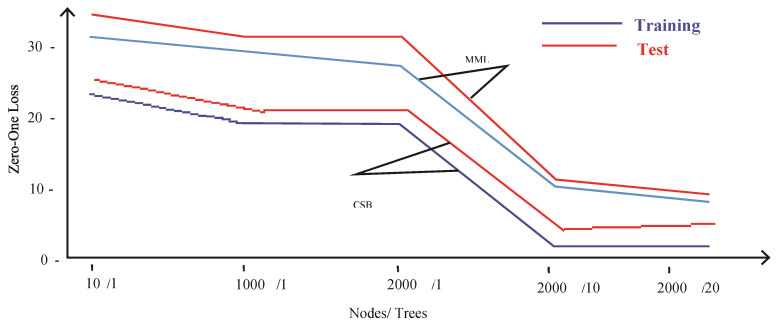
Comparison of CSB and MML approaches based on Error Loss using MNIST Dataset.

Table 1 shows that the average time taken by the MML-based model was 91.14 s, and the CSB based model was 70.33 s. Figure 7 depicts these results graphically by showing that the proposed technique is more efficient than the MML approach. The CSB approach optimal point is achieved on only 10 trees, whereas in the case of the MML approach, the number of trees is increased to 20. The MML approach error loss for training and testing is 9% and 10%, respectively, with 245 s of model building time. With the CSB approach, the error loss is reduced to only 3% and 6% for training and testing, respectively, and the model building time is reduced to 192 s.

**Figure 11:**
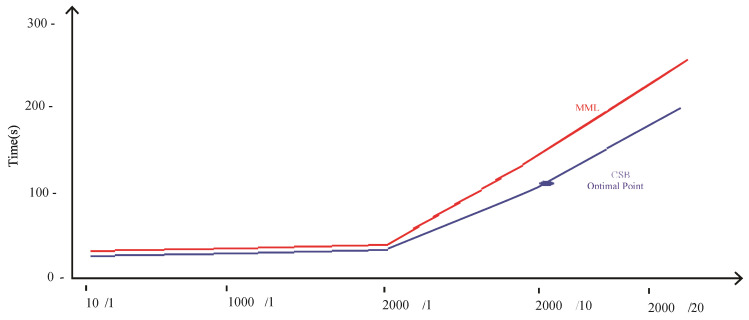
Comparison of CSB and MML approaches based Time using MNIST Dataset.

The MML and CSB models took 22.37 and 18.23 s, respectively, on tuning the parameters of 10 nodes with a single tree, as shown in Figure 11. Similarly, the MML and CSB models took 245.54 and 192.83 s, respectively, on tuning the parameters of 2000 nodes with 20 trees. These results show that, on average, the proposed technique is 20 s more efficient compared to the MML technique on the MNIST dataset.

### 3.3. Results and Comparison on Phoneme Dataset

In the Phoneme dataset [44], 3806 records were taken for training and 1621 records for testing. The aim of this dataset was to distinguish between nasal and oral vowels. Predictive models were built based on the proposed CSB and MML approaches, and both were compared on different performance parameters. The optimal value of λ for this dataset was 0.3.

**Figure 12:**
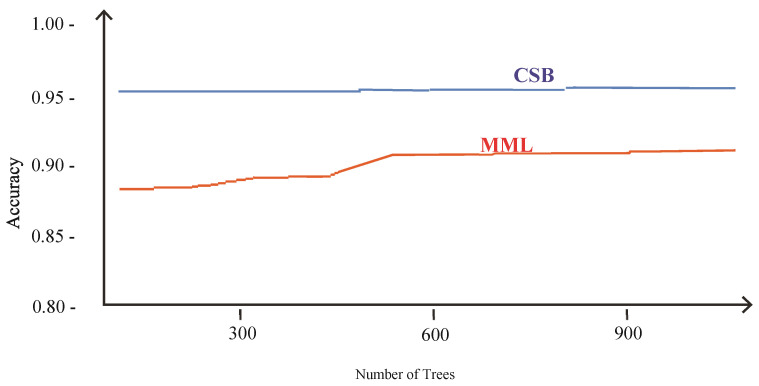
Comparison of CSB and MML approaches based on Accuracy using Phoneme Dataset.

The comparison of accuracies of MML and the proposed CSB models based on different number of trees as tuning parameters is shown in Figure 12. The maximum accuracy achieved by the MML model is 90% with 1000 trees using the Random Forest algorithm. The CSB model achieved 95% accuracy with just 100 trees. This shows that the proposed approach has achieved higher accuracy with less complexity.

**Figure 13:**
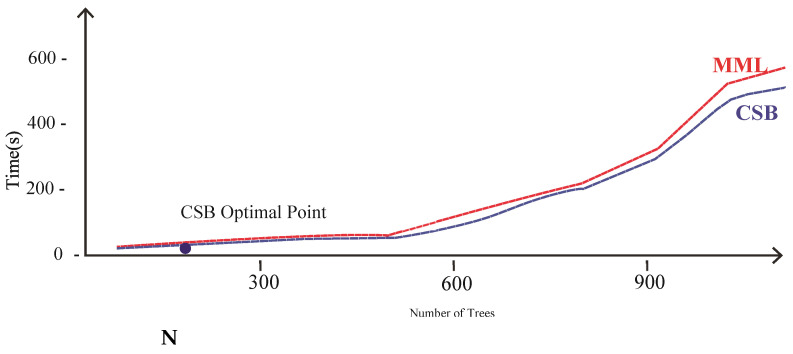
Comparison of CSB and MML approaches based on Time using Phoneme Dataset.

Figure 13 shows the execution time of the CSB and MML models. As the number of trees increases, the gap of execution time increases, which shows that the CSB model is more efficient in terms of execution time than the MML model.

## 4. Discussion and Analysis

In Section 3.1, the proposed CSB approach is compared with the MML approach on the indigenous dataset of the University of Peshwar. The results show that the maximum accuracy achieved by the MML approach is only 89% with a high complexity of 900 trees, whereas the CSB models have an accuracy of 93% with just 100 trees. This shows that the proposed approach has achieved higher accuracy with less complexity on this dataset. Similarly, the CSB models are more efficient in terms of execution time (40 s), compared to the MML model (300 s). In Section 3.2, the CSB approach is compared with the MML approach on the MNIST dataset in terms of error loss; the optimal training and testing error loss achieved using the MML approach are 9% and 10%, respectively, after tuning the number of nodes to 2000 with 20 trees, whereas, for the CSB approach, the error loss is reduced to only 3% and 6% on the tuning parameter of 2000 nodes and only 10 trees. The CSB approach is more efficient on the MNIST dataset, having an execution time of 192.83 s compared to the MML models, with an execution time of 245.54 s. In Section 3.3, the CSB approach has also improved accuracy and decreased execution time on the Phome dataset.

The above discussion shows that the model build using the CSB approach has improved accuracy and time efficiency over the MML approach. The proposed model also over performs the classical approach in terms of information lost by including the maximum complex signals and extending the fences to 1.7 × (IQR) on indigenous and Phoneme datasets, and 1.6 × (IQR) on the MNIST dataset, whereas the classical approach discards everything as outliers after 1.5 × (IQR).

## 5. Conclusions and Future Work

In the classical approach of machine learning, all data points beyond the inner and outer fences of 1.5 × IQR are considered as outliers. This leads to the loss of important information of a dataset. Models trained this way are unable to predict unseen outliers as they are not considered during training. Recently, in the modern interpolation regime, outliers are regarded as complex signals, and it is recommended that they are not avoided. The models of this paradigm do not lose important information as outliers are also considered during the training process. In this regime, overfitting is not rigidly avoided, and a model is trained even on the outliers in the dataset. The existence of a second curve (double decent curve) is observed in modern interpolation regimes by claiming that a model′s variance error starts decreasing once again after the interpolation point at the cost of higher complexity.

One problem with the modern interpolation regime is that extra training after the interpolation point results in higher execution time complexity. Another problem is the decrease in the predictive accuracy of the models. To overcome these issues, a novel preprocessing step is proposed in this research to analyze the impact of outliers’ inclusion or exclusion from the training dataset.

The performance of models is evaluated in terms of accuracy, execution time, and complexity by changing the inner and outer fences of (1.5 + λ) × IQR. Here, the λ is the fence changing parameter for exclusion or inclusion of outliers in the training dataset. The proposed approach automatically determines the optimal value of λ that includes the maximum number of outliers in the training dataset, and at the same time the model gives the best performance in terms of accuracy, execution time, and complexity. The experimental results on MNIST, Phoneme, and University of Peshawar datasets proved that the models of the proposed CSB technique outperformed the models of modern machine learning in terms of accuracy and execution time with low complexity. Future work is required to identify the optimal point for nominal attributes in the dataset. This research focused on identifying the numerical attributes in the dataset and applied the boxplot technique to perform trade off analysis on complex signals and its effect on model performance, whereas the non-numerical attributes, if they exist in the dataset, were ignored. To further improve the accuracy and performance of the model, a technique is required to additionally perform a tradeoff analysis of the non-numerical attributes.

## Figures and Tables

**Figure 8:**
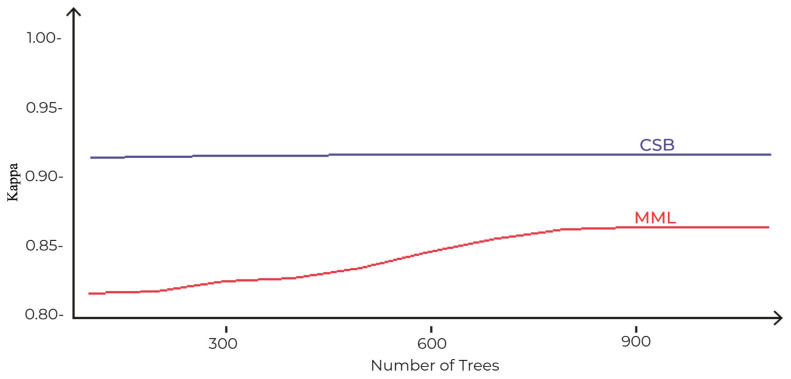
Comparison of CSB and MML approaches based on Kappa using UoP Dataset.

**Figure 9:**
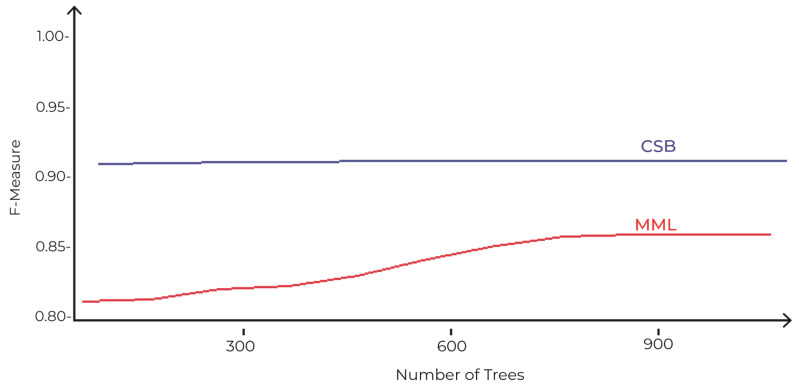
Comparison of CSB and MML approaches based on F-Measure using UoP Dataset.

**Table 1:** University of Peshawar Dataset.

Demographics	Academic Attributes																		Socio-Economic Attributes				Class
Gender	GPA	Tot_Ch	Batch	Attempts	Exam	Discipline	Roll_No	Semester	Pass Fail	Dropped	Probation	Institute	PublicPrivate	Agrade	Bgrade	Cgrade	DGrade	FGrade	HDI	Category	UrbanRural	Poverty	Grades
1	4.00	18	3	1	1	8	1	1	1	0	0	1	1	6	0	0	0	0	0.756	GS	1	50	A
1	3.98	17	2	2	2	6	5	2	1	0	0	5	1	5	1	0	0	0	0.756	GS	1	50	B
.	.	.	.	.	.	.	.	.	.	.	.	.	.	.	.	.	.	.	.	.	.	.	.
1	0.00	16	1	1	9	19	6	1	0	0	0	9	0	0	0	0	1	4	0.756	PR	1	50	F

**Table 2:** Comparison of Proposed and Modern Machine Learning approaches.

Tuning Parameters		MML			CSB		
Trees	Nodes	Training Loss Percentage	Testing Loss Percentage	Time (s)	Training Loss Percentage (λ = 0.1)	Testing Loss Percentage (λ = 0.1)	Time (s) (λ = 0.1)
1	10	32	35	22.37	24	26	18.23
1	1000	30	32	25.85	20	22	20.24
1	2000	28	32	29.68	20	22	24.31
10	2000	11	12	132.23	3	6	96.02
20	2000	9	10	245.54	3	6	192.83
Average		22	24.2	91.14	14	16.4	70.33

## Data Availability

Data will be provided on request for research purpose.
